# Navigating and Resisting Educational Systems: The Experiences of Latinx Families in Rural Oregon

**DOI:** 10.3390/bs14100863

**Published:** 2024-09-25

**Authors:** Guadalupe Díaz Lara, Tina Dodge, Soria E. Colomer, Daniel F. López-Cevallos

**Affiliations:** 1 Department of Child and Adolescent Studies, California State University, Fullerton, CA 92831, USA; 2 College of Health, Oregon State University, Corvallis, OR 97331, USA; tina.dodge@oregonstate.edu; 3 College of Education, Oregon State University, Corvallis, OR 97331, USA; soria.colomer@oregonstate.edu; 4 Department of Health Promotion and Policy, School of Public Health and Health Sciences, University of Massachusetts Amherst, Amherst, MA 01003, USA; dlopezcevall@umass.edu

**Keywords:** Latinx families, educational systems, rural communities, schools, equity

## Abstract

This study explores the experiences of Latinx parents in rural Oregon from the perspectives of Latinx parents, Latinx students, and community practitioners to understand the barriers Latinx families encounter and how they leverage their community cultural wealth to address these barriers within the context of initiatives implemented by the district. This qualitative study is guided by the following questions: What barriers do Latinx families encounter in rural schools, and how do parents leverage their community cultural wealth to address these barriers? Findings indicate that even when families were engaged in initiatives (e.g., dual language immersion programs) implemented by the district to create a welcoming environment, Latinx parents continued to encounter multiple systemic barriers (e.g., immigration laws) and leveraged multiple forms of community cultural wealth (e.g., linguistic capital) to navigate these barriers. Our findings reveal the complex dynamics at play in rural school communities serving Latinx families.

## 1. Introduction

Maintaining Latinx families’ cultural community wealth and building on their already existing strengths can facilitate Latinx families’ engagement in their children’s education. Latinx families living in rural communities across the United States have increased significantly in recent decades. In rural Oregon, 9.3% of the population identifies as Latinx, compared to 6.3% of the nation’s rural population (most from Mexican background) [1,2]. Research has revealed a myriad of challenges faced by immigrant Latinx families across geographic regions due to systemic barriers (e.g., discrimination), including challenges accessing already limited resources [3,4]. For example, Latinx families who experience discrimination at schools report lower education aspirations, which highlights the impact of discrimination and the critical role schools play in Latinx students’ and their families’ educational opportunities and experiences [5]. To increase our understanding of this issue, the present study explored the experiences of Latinx families in rural Oregon, incorporating the perspectives of Latinx parents, Latinx students, and community practitioners, which allows us to identify commonalities and differences across these groups. Additionally, we explored how Latinx families leveraged their community cultural capital (e.g., linguistic and resistance capital) to navigate the school district within the context of initiatives implemented by the district to foster a more welcoming environment. Although Latinx students make up a large percentage of students classified as English Learners (ELs) and/or students enrolled in a multilingual education program, the present study focused on students who self-identified as Latinx and were not necessarily classified as ELs or enrolled in multilingual education programs. The study is guided by the following questions: What barriers do Latinx families encounter in rural schools, and how do parents leverage their community cultural capital to address these barriers? 

## 2. Latinx Families in Rural Communities and Schools

In the last two decades, Latinx families have increasingly settled in rural “new destination” areas, located beyond traditional immigrant destination centers (e.g., Los Angeles, California; Miami, Florida) [6,7]. Rural Oregon is among these “new destination areas” with many small towns across the state experiencing a significant increase in the number of Latinx families becoming part of their communities [8]. 

### 2.1. Latinx Families in Rural Communities

In general, rural communities across the country face many challenges in supporting the education and well-being of all their residents [9]. In this context, Latinx families face additional challenges in accessing resources and navigating educational experiences. In addition to resource constraints, education and community leaders may lack the knowledge and skills to effectively support the needs of Latinx families [10] and/or the willingness and ability to tackle discriminatory practices. Often ignored discriminatory practices in rural schools can impact the well-being and educational attainment of Latinx students [10,11]. For example, Ramos and colleagues [12] found that Latinx youth in rural communities reported high levels of perceived discrimination, which in turn were associated with higher rates of depression, anxiety, and somatic symptoms. Other studies have reported similar patterns in rural communities, particularly noting the harmful impact of racism on Latinx mental health and family [13,14]. This research highlights the unique obstacles Latinx families face in rural communities, particularly when accessing educational resources, and how they leverage their community capital to navigate these challenges. 

### 2.2. Latinx Parents and Students in Schools 

Discussions of Latinx students’ educational experiences often focus on the academic achievement gap between Latinx and White students. Latinx students are often described as “lagging’’ behind their White peers and lacking the needed skills to do well in school [15,16,17]. A more critical look at these gaps, however, considers the factors that lead to opportunity gaps [18]. Previous research has documented that Latinx parents and students encounter challenges, such as difficulty navigating school policies and limited implementation of culturally and linguistically sustaining teaching practices, which can lead to high dropout rates among Latinx youth [19,20]. For example, Miranda and Rodriguez [5], found that Latinx students in rural districts in Minnesota reported significantly lower grades and were less likely to aspire to attend college than non-Latinx students in rural communities. Irvin and colleagues [21] found a slightly different pattern in a national survey, where Latinx students and their parents had similar higher educational aspirations than White rural families, but teachers had lower educational achievement expectations for Latinx students when compared to White rural students. Additionally, Latinx students reported lower ability beliefs in their academic self-concepts because of the consistent lack of high expectations from their teachers and the lack of support resources from their school districts.

### 2.3. Engaging Latinx Parents and Students

Research has also documented that when Latinx students and parents feel valued and supported by their teachers, are welcomed in the classroom, and know that their home language and culture are valued, they are academically more engaged [22]. For example, Jasis and Ordoñez-Jasis [23] found that when Latinx families felt their lived experiences, cultural knowledge, family goals, and their role as parents were respected and valued, their school engagement increased and became more meaningful. As more research emerges on strategies to engage Latinx families in schools, there is a growing realization that this will require reframing the relationships between Latinx families and school administrators, teachers, and staff to build trust and communication [24]. By valuing and leveraging the cultural capital of Latinx families, educators can develop authentic family–school partnerships, thereby fostering positive educational experiences among Latinx families [25]. Years of research have documented the practices of minoritized families who support their children’s learning by imparting the knowledge they accumulate through years of lived experiences [26]. These familial ways of knowing have been found in rural communities, where community leaders who actively advocate and leverage this knowledge have made progress in creating welcoming environments for Latinx families [27]. For example, Méndez-Morse and colleagues [28] found that educational leaders, specifically those who identified as Latinx themselves, build on the familial and linguistic strengths of Latinx families to promote meaningful family engagement in schools. Moreover, progress has been made in developing equitable and welcoming environments where leaders actively advocate and leverage Latinx families’ knowledge [27]. This study, then, contributes to our understanding of authentic family–school partnerships by exploring the types of cultural community wealth Latinx families leverage in rural contexts to navigate school settings.

### 2.4. The Present Study

This qualitative study explores the perceptions and experiences of Latinx parents, Latinx students, and community practitioners in rural Oregon, guided by the following questions: What barriers do Latinx families encounter in rural schools, and how do parents leverage their community cultural capital to address these barriers?

## 3. Methodology

### 3.1. Procedures

This study was approved by Oregon State University’s Institutional Review Board. Parents, students, and community practitioners were recruited during the school year from local schools in one rural school district in Oregon. Parents and students separately participated in focus groups, and community practitioners (e.g., teachers) participated in individual interviews. Data collection instruments were designed in consultation with members of the school district.

### 3.2. Context 

Located in rural Oregon, this school district serves almost 9000 students in the K-12 system. Within this district, 45% of the students received free/reduced lunch, 68% of the students identify as White, 22% identify as Latinx, and 10% as other. In terms of the teacher demographics, 92% identify as White, 6% as Latinx, and 2% as Asian or Multiracial [29].

### 3.3. Participants

Participation was criteria-based. Latinx parents, Latinx students, and community practitioners who met the criteria described below were invited to participate in the study. Parents’ inclusion criteria included identifying as Latinx and having at least one child enrolled in the school district. Thirty-three parents participated in the study. On average, parents were 38 years old, had 9.1 years of formal schooling, and had been living in the United States for 14.4 years. Most parents identified as Mexican (94%) and female (79%). Students who self-identified as Latinx and were enrolled in one of the secondary schools within the local school district were invited to participate. Forty-three students enrolled in middle and high schools participated in the focus groups. On average, students were 14 years old and had been living in the United States for an average of 9.8 years. They included a mix of children whose parents participated in the study and children whose parents did not participate in the study but provided consent for their children to participate. Community practitioners included those who worked with the Latinx community. Practitioners held positions such as principals, family advocates, district administrators, and health navigators. Practitioners were interviewed to explore the perspectives of those who worked or interacted with the school district and families. No demographic information was collected on community practitioners, as the focus was on the experiences of Latinx families.

### 3.4. Theoretical Framework

Drawing on the frameworks of Latinx Critical Theory [30,31] and Community Cultural Wealth (CCW) [32], we explore the experiences and perceptions of Latinx parents in a rural Oregon school district. LatCrit provides a lens to examine the intersectionality of racism, sexism, and classism, in conjunction with the linguicism and xenophobia experienced by Latinx communities in predominantly White rural communities. The present study draws on LatCrit to identify the system barriers, racism, and linguicism Latinx parents experience within this rural community and how this influences their engagement in their children’s education. Where LatCrit creates a space to name and challenge systemic racism, CCW provides a lens to (re)frame Latinx parents’ engagement with schools. 

The framework of CCW focuses on six interrelated forms of capital nurtured in communities of color [32]. These include (1) aspirational capital, which refers to parents’ ability to maintain their hopes and dreams for the future of their children in the face of real and perceived barriers; (2) linguistic capital, which refers to parents’ ability to develop communication skills through various experiences; (3) familial capital, which refers to the social and personal human resources parents have in their environment from extended family to community networks; (4) social capital, which refers to peers and social contacts and how parents utilize those contacts to navigate social institutions; (5) navigation capital, which refers to parents’ skills and abilities to navigate social institutions; and (6) resistance capital, which refers to the experiences of parents in securing equal rights for their children and families [32]. In the present study, we draw on CCW to examine how Latinx parents leveraged their various forms of capital to address the barriers their families encountered in a rural school district. 

### 3.5. Data Collection 

Parents, students, and community practitioners were recruited during the school year from local schools in one rural school district in Oregon. All parents and students completed a demographic survey. Questions included demographic information such as level of education, age, and gender. Parents and students separately participated in focus groups, and community practitioners (e.g., teachers) participated in individual interviews. Participants were not compensated for taking part in the study. 

#### 3.5.1. Focus Groups and Interviews

Parents self-selected to participate in one of five focus groups. There were 4–6 participants in each parent focus group, and all were conducted in Spanish. Students also self-selected to participate in one of four focus groups. Each student focus group had 7–10 participants. Students used a combination of English and Spanish during the focus groups to express themselves, because they were instructed to use whichever language they felt more comfortable with. The focus groups with parents and students lasted between 30 and 60 min and were conducted by a bilingual and bicultural researcher. Community practitioners (e.g., teachers) participated in individual interviews. Interviews were conducted in English or Spanish depending on the language preference of each participant. Focus groups and interviews addressed three areas: (1) basic needs that interfered with the academic success of Latinx students; (2) parents’ and students’ experiences with the schools; and (3) school-based resources and services needed to support the academic achievement of students. The interviews lasted between 30 and 60 min and were conducted by the first author, who is a bilingual and bicultural researcher (See Appendix A).

#### 3.5.2. Demographic Surveys

All parents and students completed a demographic survey. Questions included demographic information such as level of education, age, and gender.

### 3.6. Data Analysis

All focus group and interview data were transcribed and uploaded into NVivo, a data management system [33]. Focus group and interview transcripts were analyzed by the authors in their original language with descriptive coding, which assigns a label to the data by using a word or phrase to represent the data with a combination of inductive and deductive approaches to develop a coding system across focus group and interview data [34]. The first step in the coding process was to read over individual transcripts, including transcripts from the parents’ and students’ focus groups and practitioners’ interviews, and to create potential codes individually. After the initial review of transcripts, the second step was for the coding team to discuss potential codes and create a preliminary codebook with definitions and examples from the data. The third step was to test the codes in 10% of the focus group and interview transcripts to ensure codes were applied consistently among team members. Using 10% of the transcripts, the fourth step was to conduct a percentage agreement analysis among all codes to ensure at least 80% agreement. 

The percentage agreement among coders ranged from 85–98% across codes. After the percentage agreement among codes reached above 80%, the codebook was considered finalized, and the final step was for transcripts to be divided among coders to code individually. A total of 7 categories were created, and each category had 1–6 individual codes for a total of 20 individual codes across all the transcripts. To ensure ongoing consistency among coders, the coding team met weekly to discuss any questions and/or challenges during the coding process [35]. Codes are summarized below and are represented by quotes from the data [34]. Quotes in Spanish were translated by the first author and are reported in Spanish and English. 

### 3.7. Team Positionality Statement

During our research process, the team reflected on their social identities and how these might influence our research process as part of our weekly meetings. Our research team either self-identifies as Latinx or as an ally of (with a long history of working with) the Latinx community. We have extensive experiences both personally and professionally within the Latinx community. Discussing the influence of our social identities helped us to incorporate the influence of these identities in our research process. 

## 4. Results

The school district implemented programs and strategies to foster a welcoming environment for Latinx families. However, findings demonstrate the complexity of creating a welcoming environment given the experiences of Latinx families in this rural community. The following section illustrates the efforts taken by the district to foster a welcoming environment, the barriers Latinx families encountered, and actions taken by Latinx families to navigate barriers in a rural school district. 

### 4.1. Culturally and Linguistically Sustaining Programs and Practices

#### 4.1.1. Dual Language Immersion Programs and Spanish Classes

In the early 2000s, the school district implemented a Spanish–English Dual Language Immersion (DLI) program at one elementary and one middle school that served the majority of the Latinx families in the district. Additionally, the district offered Spanish classes for heritage speakers at the high school level for students who wanted to continue learning Spanish. At the time of the study, there was no official pathway for students to continue to develop their Spanish language skills beyond these courses. One parent mentioned, “Que tengan los dos idiomas—eso me encanta. Que [el programa] es bilingüe es algo que me gusta bastante.” [That they have both languages—I really like that [DLI program] is bilingual… it is something that I like a lot]. In addition, one teacher recommended that more upper-level Spanish courses be offered at the high school for students to continue building their language skills. A high school principal echoed this sentiment and underscored the benefits of developing Latinx students’ home language. He noted,

“We’re now offering courses that allow those students to build on their skills rather than say, ‘Well you can’t take Spanish because you already know it, so go take German instead.’ I mean, that’s great if they want to [take German] but we could also really enhance their Spanish skills.”

Although multiple stakeholders agreed that DLI programs and Spanish classes created a welcoming school environment, Latinx parents were still left out when making decisions related to their children’s academic progress. 

#### 4.1.2. School–Family Communication

Conflicts about when Latinx parents were notified or involved in the decisions made about their children’s academic trajectories often arose between Latinx parents, students, and school staff. Latinx parents and students felt that parents were not informed when children struggled academically or when behavior concerns arose. This was especially the case for children who were in middle school and high school. Participants explained that there was a difference in beliefs around children’s independence between parents and school administrators. Secondary schools promoted values of independence and encouraged students to manage their own learning. Values promoted at school, then, were at odds with the values of Latinx families who strongly emphasized the importance of supporting children during adolescence. This sentiment was shared by students and some of the staff who worked directly with Latinx families. One principal noted the conflicting message educators sent parents once their children entered middle school, “We tell parents, ‘Please come help us in grade school’ and then as we transition into middle school and high school and we say, ‘Parents, please stay away’”. 

Latinx parents emphasized that it was their responsibility to motivate their children and to find resources that addressed academic challenges; it was not something children should have to do on their own. Additionally, parents associated teachers’ lack of transparency about their children’s academic performance with teachers’ low educational expectations for Latinx children. One parent noted,

“Los padres tenemos altas expectativas para nuestros hijos y yo creo que eso es algo que los maestros tienen que saber, porque mi hija es latina y habla español, no quiere decir que mi hija no va ir a la universidad … o sea que sean transparentes y que digan, mira sabes a su hija le falta, necesita ayuda, yo sé que ellos quieren verlo de una manera positiva, pero realmente los padres no lo entendemos así, lo vemos mal.” [As parents we have high expectations for our children, and I think that is something teachers should know because my daughter is Latina and she speaks Spanish, that does not mean that my daughter is not going to go to the university … be transparent and say, ‘Look, your daughter is not doing well, she needs help.’ or whatever it is. I know they want to frame it in a positive way, but in reality, as parents, we do not see it that way; we see it as wrong].

Latinx families often leveraged their aspirational capital to navigate academic expectations. Parents expressed that it was their responsibility to keep their children motivated about their education and future, as children often did not make the best decisions for themselves. Additionally, parents used their navigational capital to maneuver through social institutions and access resources to support their children’s academics. When parents asked for support from teachers and school administrators, and the support was not provided, parents searched for services and support outside the school. Securing academic resources for their children regardless of the challenges was non-negotiable for parents. One parent expressed,

“Me dieron dos semanas, él no estaba a nivel de segundo lo iban a bajar otra vez pues a primero, pues yo me puse las pilas para buscar un tutor y ya cuando entro sí lo pusieron a prueba y el ya iba avanzado.” [They gave me two weeks for him to be ready for second grade. If not, they would keep him in first grade. So I found a tutor, and when he went back, they tested him, and he was making progress].

#### 4.1.3. Translation and Interpretation Services

Bilingual/bicultural staff were hired in some schools to facilitate communication with parents. One mother shared,

“Me gusta mucho porque tienen mucho personal bilingüe… si no puedo llenar papeles voy a la oficina y siempre hay alguien quien me ayude.” [I like it a lot because there are a lot of bilingual staff… if I am unable fill out documents, I go to the office, and there is always someone to help me].

Access to translation and interpretation services supported families’ ability to engage with their children’s school by facilitating communication. However, these services were not available to all families and were often inconsistent or of low quality. As a result, Latinx parents were unable to connect with the bilingual/bicultural staff. Hence, many Latinx parents remained unheard of and felt unwelcome in school settings. As one parent described,

“En la escuela de mi hijo dicen que hay una persona que habla español, pero cada vez que voy no que no está, yo no creo que haya alguien que hable español.” [At my child’s school, they say there is a person who speaks Spanish, but every time I go, nobody is there, so I don’t think there is a person who speaks Spanish].

Parents recalled their experiences when trying to engage at a Parent Teacher Organization (PTO) meeting; one shared,

“Una vez fui, pero como que la mitad entendí y la otra no. Pues, me salí … antes que se terminara porque no entendí mucho. Así que dije, ‘Mejor me voy.’ Y ya no regresé.” [I went once, but I understood about half, and the other half I did not. Well, I left before the meeting ended because I didn’t understand much. So I said, ‘I better leave.’ And I did not go back].

Adding to the inconsistency of these services, Latinx families also commented on the quality of services and the loyalty of the interpreters. Often, parents mentioned that the interpreters did not translate exactly what they were saying to district staff, especially if there was a complaint and/or a concern being discussed. Latinx parents noted that the lack of accurate translation was likely because the staff who provided translation and interpretation services worked for the district, and they were there to serve the district. 

Students also noted the need for parents to communicate with teachers. One student shared, “My mom does not feel comfortable talking with the staff … she does not connect with the staff, and it’s very important because sometimes kids need their parents, and they [parents] need to talk with school”, referring to the importance of the school facilitating translation services for parents.

While all participants indicated the importance of having high-quality and consistent translation and interpretation services, Latinx parents made multiple efforts to develop their linguistic capital by taking courses to learn English and use their English language to communicate with teachers. For example, one parent indicated, “Yo hablo inglés pero no lo suficiente y trato de estudiar, como mirar dónde dan clases. Yo voy y así voy aprendiendo poco a poco.” [I speak English, but not enough, so I try to study and look for where classes are taught, and I learn little by little]. Although the district was aware of some of the barriers families experienced, they did not seem to know how to effectively address them and did not prioritize the intentional allocation of resources to consistently implement strategies to reduce their impact. 

### 4.2. Community Partnerships

#### 4.2.1. District Community Center

Many Latinx parents felt disrespected when they entered social service offices. When Latinx families tried to access health services, interpreters were hard to find, contributing to a sense that agencies were making little effort to understand and address their needs. One health navigator pointed out, “There is no interpretation available to help you, also the paperwork is a lot. I think the community is working on it, but the information is not in Spanish, and, if it is in Spanish, it is not translated correctly.” To make these services easier to access for Latinx parents, the district created a community center. The community center was centrally located and had interpreters available to help Latinx parents access school services and resources beyond school-related programs. The community center connected families with social services (e.g., health insurance), offered educational workshops (e.g., learning how to navigate the school system), and housed KidsShop (i.e., a program that provided clothing and school supplies for students). One student noted,

“El centro te da lo que tu necesitas. Por ejemplo, cuando llegas aquí no tienes absolutamente nada, también te dan mochilas, y cosas, y también útiles escolares que vas a ocupar aquí, también te ayudan a inscribirte a la escuela, cuando vas ingresando apenas, te ayudan mucho.” [The Center gives you everything you need. For example, when you get here and you have absolutely nothing, they give you backpacks and other things, and school supplies you are going to need. They also help you register for school when you are starting. They help you a lot].

Many parents considered the community center key to engaging with the school and supporting their children’s education, but many parents still did not know about the services offered by the community center. 

#### 4.2.2. Dissemination of Information and Access to Services

The school district made efforts to connect families to community services to support families’ needs, but often families were not aware that these services existed. As one parent noted, “hay muchas ayudas pero … los padres no están informados de la ayuda; el Centro es muy bueno pero yo he preguntado… y mucha gente ni siquiera sabe que existe.” [There are a lot of services, but parents are not well informed about the services; the Center is great, but I have asked… and many people do not even know it exists]. While the district made efforts to disseminate information, these efforts were not effective at reaching Latinx families. For example, the district relied on flyers and emails to disseminate information, whereas Latinx families relied on social networks. Based on Latinx families’ experiences, it was not enough for the school district to provide services, as these resources did not benefit Latinx families who were unaware of the services available to them and their children. 

To access information and services parents often relied on their social capital. One parent who was aware of services at the Community Center mentioned, “en cuanto acabemos aquí vamos a que la señora nos enseñen dónde están.” [as soon as we are done here, we are gonna go with the lady, so she can show us where they are]. In addition to this instance, parents shared multiple examples of social capital within the Latinx community that helped them connect to resources and understand how the school system functioned. 

### 4.3. Systemic Racism, Discrimination, and Policies

Although the school district was making efforts to support families, there were multiple systemic barriers that kept families from being fully involved in their children’s education. These experiences are described below. 

#### 4.3.1. Discrimination and Racism

Latinx families often experienced discrimination and racism. There were multiple examples of the discrimination and racism that Latinx families experienced living in a predominantly rural White community. These experiences ranged from racist symbols around the community to the curriculum in the classroom. For example, the English Language Learner Specialist noted,

“It’s just [a] very White community…one of our high school mascots is the rebel soldiers from the civil war and I see trucks driving around flying the confederate flag. It just amazes me …I just think the district could do more to educate all employees, all staff on cultural diversity and even go as far as to help people recognize what privilege is…what is privilege? What bias do you have? What is the effect on the families and students that you’re serving? I think a lot of people don’t realize and have a kind of unconscious bias; we all have it.”

Latinx students described similar experiences in the classroom, especially when the curriculum included immigration and US/Mexican history. Students added that they did not experience overt racism but rather covert racism. Below is an excerpt from a student focus group when asked about their experiences at school.

Student 1: “It’s like you never really face it ‘til in, you know, in class and then somebody you know you think you’re cool with and then they’re like all illegals (“Illegal(s)” is a derogatory term used in the United States to refer to those who do not have a resident card or citizenship and live in the United States.) should be deported.”

Student 2: “And there’s a lot of people that feel like that at this school.”

Student 1: “It’s not like actively like, ‘Oh, get out of here. Don’t come to this school.’ It’s more like a passive kind of thing.”

Latinx families often leveraged multiple forms of capital to mitigate the racism and discrimination their families experienced. Participants explained how their familial capital and social capital helped them to identify safe spaces within the community. One student noted when describing places where others identify as Latinx even if they did not personally know each other,

“I think it’s just the comfort level, you know everyone, well not just that like you can know everybody at some place, but you don’t feel the same going to school with a bunch of like different people than you. Then it’s so hard to say it; I don’t know how to explain it, actually. It’s where you feel comfortable.”

Families also leveraged their resistance capital by challenging the stereotypes that dehumanized their communities and the way these impacted their children. One parent explained,

“Reconocerles a ellos que no soy un nopal, no son un sombrero… que ellos son importantes para la comunidad, porque cuando vamos a la tienda mexicana o cuando vemos esos banderines… mira eso es nuestro símbolo, eso es lo que representa nuestra comunidad … nuestra cultura.” [recognize them, they are not a cactus, they are not a hat… they are an important part of the community, and when we go to the Mexican store or when see the flags… that is our symbol that represents our community … our culture]

#### 4.3.2. Immigration Policies

In addition to the racism and discrimination parents encountered, they also feared the consequences of anti-immigration laws (e.g., deportation, family separation). The inability to obtain a driver’s license (A driver’s license is required in the United States to drive. To obtain a driver’s license in the United States, a person must pass a knowledge driving test and a driving test and present identification documents. In some states, identification documents include proof of being authorized to live in the United States.), for example, worried all parents and was one of the main barriers that prevented parents from fully engaging in their children’s education (e.g., unable to drive their children to school). A parent shared his concerns, “Las necesidades es la licencia, no nos permiten manejar seguros aquí, nosotros a veces, las personas manejamos por necesidad muchas veces no por gusto… es una necesidad grandísima que tenemos nosotros aquí en la comunidad.” [The need is the driver’s license. We are not allowed to drive safely here…We drive out of necessity, many times, not for pleasure… It is a huge need that we have in our community]. Another parent shared how not having a driver’s license impacted their mental health and, as a result, their interactions with their children. One parent shared, “no se puede manejar o cuando vas manejando vas con un miedo y con un estrés que te cambia completamente el modo.” [You can’t drive, or when you are driving, you are so scared and stressed that it completely changes your mood]. Their inability to obtain driver’s licenses impacted everything from mental health to job opportunities that, in the end, impacted their ability to engage with their children’s education. This parent noted,

“Todo está conectado, no puedes trabajar legalmente, no puedes manejar legalmente, no puedes hacer muchas cosas y yo veo a los padres que llegan deprimidos, frustrados… y llegan los niños con las tareas… ayúdanos con la tarea, o sea cómo?” [Everything is connected. No, you cannot work legally, you cannot drive legally, you cannot do things, and I see the parents, they arrive depressed, frustrated… then kids arrive with homework… Help us with homework, I mean, how?]

Latinx families leveraged their resistance capital and challenged the inequality that the lack of a driver’s license brought by continuing with their lives and raising awareness of the impact of not having a driver’s license. One parent noted,

“En mi caso van hacer como dos años que no tengo licencia… la vida sigue yo tengo que llevar mis hijos a la escuela, tengo que llevar a mis hijos al doctor, tengo que ir al doctor para mi, para mi mamá, y no quiere decir que no tienes licencia no puedes manejar, tu vida se va a parar ahí claro que no, mi vida sigue y yo sé el riesgo”. [In my case, it is going to be two years since I do not have a license… life keeps going, I have to take my kids to school, I have to take my kids to the doctor, I have to go to the doctor for me, for my mom. It does not mean that because you do not have a license, you cannot drive, or your life is going to stop there, of course not. Life keeps going, and I know the risk].

Not being able to obtain a driver’s license also directly impacted parents’ ability to volunteer in the schools, given that public transportation is not readily available or reliable in rural communities. Additionally, when parents completed their volunteer application, it specifically asked for a state driver’s license or identification. The school district was unwilling to accept other forms of identification, such as a consular registration that parents would be more likely to obtain from their country’s consulate offices. From parents’ perspectives, having to provide this documentation created an environment of fear because parents were afraid of the consequences of the school finding out they were undocumented in the US. A parent noted how the school could utilize volunteer applications to ascertain immigration status and keep parents out of the school. One parent noted, “me pidieron el ID pero seguro que te están pidiendo el estatus migratorio porque te lo disfrazan del ID.” [they asked me for an ID, but surely, they are asking you for your immigration status because they disguise it as your ID].

Latinx families leveraged their social capital and their familial capital to counteract the impact of not being able to apply for a driver’s license. Often, Latinx families relied on their social network to drive them or their children to places. When talking about driving, one parent explained, “le pido a otras personas lo más que puedo” [I ask other people as much as possible], but this was a temporary solution. Additionally, parents had to rely on their family, specifically, their high school children who were able to get driver’s licenses. Although parents did not like this solution because it placed unnecessary responsibility on their children, their children took up this responsibility because they understood the detrimental consequences of their parents driving without a license. 

## 5. Discussion and Implications

The purpose of this study was to explore the experiences of Latinx families in a rural school district in Oregon from multiple perspectives (students, parents, and community practitioners). As illustrated by the findings, the district made efforts to create a welcoming environment for Latinx families, but there were still a lot of action steps, awareness, reflection, and intentionality that needed to occur within the district to effectively serve Latinx families and leverage their cultural capital to support students’ learning within a rural context. 

Drawing on a LatCrit theoretical framework allowed us to center the impact of policies and practices that were rooted in systemic racism and classism in combination with linguicism and xenophobia, which Latinx families experience in this predominantly White rural community. These racist policies and practices resulted in systemic barriers for Latinx families to engage in their children’s learning and access resources. For example, the unwillingness of the district to accept alternative forms of identification when residents were unable to legally obtain driver’s licenses, along with the lack of professional development for teachers, staff, and administrators on systemic oppression and teaching pedagogy to support Latinx students and their families, contributed to the challenges faced by these families. Additionally, drawing on CCW (Community Cultural Wealth) as a framework to understand Latinx parents’ responses highlighted the multitude of community strengths Latinx families leverage daily [32]. Latinx families do not passively experience barriers, but rather actively resist the policies and practices that created barriers for them to engage in their children’s learning. This aligns with the previous literature indicating that Latinx families in rural communities use a variety of assets and resources to navigate and resist the challenges they experience [36]. The experiences shared by Latinx parents, students, and practitioners in our study illustrate the complexity of creating effective partnerships with Latinx families, as well as the potential actions rural districts must take to promote community cultural wealth among Latinx families and address systemic oppression (e.g., racism) to create a welcoming environment. As suggested by Gallo and colleagues [37] in their work with Latinx families in the suburbs, such an approach requires expanding the definition of parent engagement to include the cultural capital that families use to both navigate challenges in their local communities and contribute to their children’s education. Instead of overlooking Latinx parents’ cultural capital, districts would better serve Latinx families by engaging in authentic conversations on how they can address systemic barriers and leverage families’ cultural capital to create a welcoming environment. 

Additionally, the district can make intentional efforts to implement and prioritize programming that previous research has documented as an effective way to nurture community cultural wealth. For example, the DLI program promoted linguistic capital, and previous research has shown that this is a high-quality culturally responsive programming that can be an effective strategy for creating a welcoming environment for Latinx families and nurturing community cultural wealth [38,39]. To continue to leverage the linguistic capital of Latinx families, the school district could expand their DLI program to other schools as well as their middle and high schools to provide opportunities for students to continue to build on their home language. 

Although the district implemented practices to foster a more welcoming environment, there were additional barriers that limited the impact of these practices. For example, the district provided translation services, but these were often inconsistent and of low quality. To support navigational capital, the district can intentionally allocate funding to provide high-quality and consistent translation services, so families are able to focus on supporting their children’s educational needs instead of finding adequate ways to communicate. The district also had programs that supported families, but families did not know about the available services or how to access them. It was not as simple as just providing the services or creating programs for families, especially if families were not aware of the services and resources available and if these services were inconsistent. For example, the community center was an asset to the community, but the district had not implemented effective strategies to inform Latinx families of the services offered. This is consistent with other findings that illustrate that to effectively engage Latinx families in schools, there needs to be an understanding of the needs and respect for the strengths of Latinx families [40,41,42]. The school district could leverage Latinx families’ social capital to ensure families are aware of the services provided by the district. This can mean partnering with local parent groups or organizations that have a direct and trustworthy collaboration with Latinx families. 

Families leveraged their community cultural capital to make up for inconsistent and insufficient resource dissemination from the district. The time and effort expended by Latinx parents who tried to make up for what the school district was supposed to provide could have instead been directly invested in their children (e.g., finding extra-curricular activities, participating in parent leadership programs.). Although Latinx families managed to address barriers, the district did not prioritize or leverage the families’ existing cultural capital to support learning. 

A critical missing piece in the practices and programs implemented by the school district was that none of these efforts included understanding how systemic barriers rooted in racism and discrimination impacted Latinx families. For example, there was often a lack of understanding from the district staff on how immigration policies kept Latinx parents from obtaining driver’s licenses. There were no professional development opportunities for administrators, teachers, and staff to learn and reflect on the ways systemic barriers impacted the learning and well-being of students and parents. Although undocumented adults are now able to obtain a driver’s license in Oregon (a new law allowing them to obtain driver’s licenses came into effect on 1 January 2021) [43], findings from the present study can inform other states where undocumented immigrants are still unable to receive a driver’s license. The school district can implement professional development efforts district-wide that prioritize understanding systemic barriers and racism using a LatCrit framework to effectively support Latinx students and families and create welcoming environments. 

Our findings are consistent with research that illustrates the challenges Latinx families encountered as they navigated school systems [25]. Additionally, our findings contribute to existing research that challenges the deficit notion that Latinx families are not involved or do not want to be involved in their children’s education [22,44], which seems to be particularly pervasive in rural communities [45]. We demonstrate the multiple forms of capital that parents leverage to provide their children with high-quality education and promote the well-being of their family by finding needed resources, while navigating racism, linguicism, and xenophobia [22,46,47]. As we further our understanding of the complexities of effectively engaging Latinx families, it is crucial that school districts create and expand programs (such as the DLI—Dual Language Immersion program) as potential strategies to create welcoming environments, leverage Latinx families’ cultural wealth, make continuous and consistent efforts to effectively communicate with Latinx families, disseminate information on resources available, provide training on how to access these resources, and train district personnel on cultural competency professional development to more effectively engage Latinx families. 

## 6. Limitations

The present study has several limitations. First, our parent and student study participants were of Mexican background. Therefore, it is possible that that our study is not reflecting the experience of Latinx families of other backgrounds. Future studies should explore the experiences of Latinx families from other Central and South American countries in rural communities, as well as explore how the intersectionality of social identities (e.g., immigration status, language, socioeconomic status) might influence these experiences. Second, the rural town where the data was collected is in the Pacific Northwest, and the experiences of Latinx families in rural communities in other regions of the U.S. may vary. Therefore, it would be beneficial to explore these issues in other regions of the U.S. to broaden our understanding of the experiences of Latinx families in rural communities. Third, English Learner (EL) status is a key issue for Latinx families that we were unable to explore in the present study. Future studies should look specifically at the schooling experiences of Latinx students who are also identified as ELs in rural communities. Nonetheless, the present study provides a deeper understanding of the barriers experienced by Latinx families and the community cultural capital that Latinx families leverage to navigate rural communities. 

## 7. Conclusions

Findings from the present study highlight the complexity of creating welcoming environments for Latinx families that nurture and leverage community cultural wealth. Continuing the discussion on the experiences of Latinx families in rural communities can support efforts to keep their needs at the forefront as programs, practices, and policies are designed, implemented, and evaluated in rural communities across the country. 

## Data Availability

Data are contained within the article.

## Appendix A. Focus Group and Interview Guides

### Appendix A.1. Focus Group Guide

Do you consider this community a good place to live? Please explain.

1. In general, what has been your experience with schools?
  - What do you like the most/the least?
2. Are there basic family, child, and youth unmet needs that may affect Latino students’ educational attainment?
3. What would it take for (your) parents to be more involved in schools? (e.g., interpreters, bilingual/bicultural staff)
4. What is your experience in school? Have there been any changes? How could schools be more supportive? How could schools better support Latino students and their parents?
5. How could schools better support Latino families to address unmet needs that affect their children’s educational attainment?
  - Are there other organizations that could support Latino families with these issues?

### Appendix A.2. Interview Guide

1. Do you consider [community] a good place to live for Latino families? Please explain.
2. In general, what is your perspective regarding the educational experience for Latino families in [community] schools?
  - What are the strengths/weaknesses?
  - Educational attainment?
3. What would it take for parents to be more involved in schools? (e.g., interpreters, bilingual/bicultural staff)
4. How could schools better support Latino students and their parents?
5. How could schools better support Latino families to address unmet needs that affect their children’s educational attainment?
  - Are there other organizations that could support Latino families with these issues?

## References

[1] The Oregon Community Foundation (2016). Latinos in Oregon: Trends and Opportunities in a Changing State. https://oregoncf.org/Templates/media/files/reports/latinos_in_oregon_report_2016.pdf.

[2] US Census Bureau (2021). 2013–2017 American Community Survey 5-Year Estimates. https://www.census.gov/programs-surveys/acs.

[3] Burke M.M. (2017). Examining empowerment, family–school partnerships, and advocacy among rural and urban Latino families of children with disabilities. Rural. Spec. Educ. Q..

[4] Miller A.L. (2019). (Re)conceptualizing family-school partnerships with and for culturally and linguistically diverse families. Race Ethnicity Educ..

[5] Miranda A., Rodriguez M.C. (2022). Contexts of educational aspirations and school grades of rural Students. Russell Sage Found. J. Soc. Sci..

[6] Stein G.L., Gonzales R.G., García Coll C., Prandoni J.I., Crockett L.J., Carlo G. (2016). Latinos in rural, new immigrant destinations: A modification of the integrative model of child development. Rural Ethnic Minority Youth and Families in the United States: Theory, Research, and Applications.

[7] Terrazas A. (2011). Immigrants in New-Destination States. Migrant Policy Institute. https://www.migrationpolicy.org/article/immigrants-new-destination-states.

[8] López Salinas A., Curiel-Avilés U.G., Reyes Morales R.G. (2016). Engaging Mexican Populations in New Immigrant Destinations: Mexicanos in Oregon. Int. J. Community Divers..

[9] Crumb L., Chambers C., Azano A., Hands A., Cuthrell K., Avent M. (2023). Rural cultural wealth: Dismantling deficit ideologies of rurality. J. Multicult. Educ..

[10] Means D.R., Sansone V.A., Azano A.P., Eppley K., Biddle C. (2021). Latinx students in rural schools. The Bloomsbury Handbook of Rural Education in the United States.

[11] Gándara P., Gutiáez D., O’Hara S. (2001). Planning for the Future in Rural and Urban High Schools. J. Educ. Stud. Placed Risk (JESPAR).

[12] Ramos G., Ponting C., Bocanegra E., Chodzen G., Delgadillo D., Rapp A., Escovar E., Chavira D. (2022). Discrimination and Internalizing Symptoms in Rural Latinx Adolescents: The Protective Role of Family Resilience. J. Clin. Child Adolesc. Psychol..

[13] Lichter D.T., Parisi D., Taquino M.C. (2016). Emerging Patterns of Hispanic Residential Segregation: Lessons from Rural and Small-Town America. Rural Sociol..

[14] Parmar D.D., Minnis A.M., Caballero E., Zerofsky M., Comfort M., Raymond-Flesch M. (2023). Latina mothers’ perspectives on adverse experiences and protection of Latinx youth in an agricultural community. BMC Public Health.

[15] Gándara P., Contreras F. (2010). The Latino Education Crisis: The Consequences of Failed Social Policies.

[16] López N. (2000). The Missing Link: Latinos and Educational Opportunity Programs. Equity Excell. Educ..

[17] Reese L., Jensen B., Ramirez D. (2014). Emotionally supportive classroom contexts for young Latino children in rural California. Elem. Sch. J..

[18] Kohli R., Pizarro M., Nevárez A. (2017). The “new racism” of K–12 schools: Centering critical research on racism. Rev. Res. Educ..

[19] Gonzales-Berry E., Mendoza M. (2010). Mexicanos in Oregon: Their Stories, Their Lives.

[20] Lopez Green V., Vargas Poppe S. (2021). Toward a More Perfect Union: Understanding Systemic Racism and Resulting Inequity in Latino Communities. Unidos US. https://www.unidosus.org/wp-content/uploads/2021/08/unidosus_systemicracismpaper.pdf.

[21] Irvin M.J., Byun S., Meece J.L., Reed K.S., Farmer T.W. (2016). School characteristics and experiences of African American, Hispanic/Latino, and Native American youth in rural communities: Relation to educational aspirations. Peabody J. Educ..

[22] Jasis P. (2021). Latino immigrant parents and schools: Learning from their journeys of empowerment. J. Lat. Educ..

[23] Jasis P.M., Ordoñez-Jasis R. (2012). Latino parent involvement: Examining commitment and empowerment in schools. Urban Educ..

[24] Coady M.R., Coady T.J., Nelson A. (2015). Assessing the needs of immigrant, Latino families and teachers in rural settings: Building home-school partnerships. NABE J. Res. Pract..

[25] Arellanes J.A., Viramontez R.P., Lohman B.J. (2018). Bettering the educational attainment for Latino families: How families view the education of their children. J. Lat. Educ..

[26] Maldonado Torres S.E. (2022). Demystifying Latinos parental involvement in school activities. J. Lat. Educ..

[27] Viramontez Anguiano R., Martinez M., Chavez J., Harrison S.M., Bellowe J.J. (2022). Pueblos y Ranchos: The role of Latino fathers and community leaders in serving Latino families in rural Northwest Iowa and Southern Minnesota. J. Fam. Strengths.

[28] Méndez-Morse S., Murakami E.T., Byrne-Jiménez M., Hernandez F. (2015). Mujeres in the principal’s office: Latina school leaders. J. Lat. Educ..

[29] Oregon Department of Education (2021). At-A-Glance Profiles for Schools and Districts. https://www.oregon.gov/ode/schools-and-districts/reportcards/reportcards/pages/default.aspx.

[30] Solórzano D., Yosso T. (2001). Critical race and LatCrit theory and method: Counter storytelling Chicana and Chicano graduate school experiences. Int. J. Qual. Stud. Educ..

[31] Solórzano D., Delgado Bernal D. (2001). Critical race theory, transformational resistance and social justice: Chicana and Chicano students in an urban context. Urban Educ..

[32] Yosso T.J. (2005). Whose culture has capital? A critical race theory discussion of community cultural wealth. Race Ethn. Educ..

[33] QSR International Pty Ltd (2020). NVivo (Released in March 2020). https://www.qsrinternational.com/nvivo-qualitative-data-analysis-software/home.

[34] Saldaña J.M. (2021). The Coding Manual for Qualitative Researchers.

[35] O’Connor C., Joffe H. (2020). Intercoder reliability in qualitative research: Debates and practical guidelines. Int. J. Qual. Methods.

[36] Raffaelli M., Wiley A.R. (2013). Challenges and Strengths of Immigrant Latino Families in the Rural Midwest. J. Fam. Issues.

[37] Gallo S., Wortham S., Bennet I., Hamann E., Wortham S., Murillo E. (2015). Increasing ‘Parent Involvement’ in the New Latino Diaspora. Revisiting Education in the New Latino Diaspora.

[38] DeMatthews D.E., Izquierdo E. (2020). Leadership for social justice and sustainability: A historical case study of a high-performing dual language school along the U.S.-Mexico border. J. Educ. Stud. Placed Risk.

[39] Prado Y., Ramos M.N., Peña E., Zavala J. (2022). Dual-language engagement: Concerted cultivation of Spanish use among students, teachers, and parents. Biling. Res. J..

[40] Cavanagh T., Vigil P., Garcia E. (2014). A Story Legitimating the Voices of Latino/Hispanic Students and their Parents: Creating a Restorative Justice Response to Wrongdoing and Conflict in Schools. Equity Excell. Educ..

[41] Lowenhaupt R. (2014). School Access and Participation: Family Engagement Practices in the New Latino Diaspora. Educ. Urban Soc..

[42] Díaz Lara G. (2024). Liberal policies does not mean equitable policies: Latinx families’ experiences navigating social policies in Oregon and California. Cult. Divers. Ethn. Minor. Psychol..

[43] Oregon Driver & Motor Vehicle Services (2022). Driver Licenses for All (HB2015). https://www.oregon.gov/odot/dmv/pages/hb2015.aspx.

[44] Moll L.C., Soto-Santiago S.L., Schwartz L., Hall K., Cremin T., Comber B., Moll L.C. (2013). Funds of knowledge in changing communities. International Handbook of Research on Children’s Literacy, Learning, and Culture.

[45] Goedeken J.A., Xia Y., Durden T., de Guzman M.T. (2016). Rural Hispanic Youths’ Perceptions of Positive Youth Development Experiences. J. Ext..

[46] Espinoza K., Degollado E.D. (2023). “You Just Want to Start Trouble”: A Chicana Bilingual Maestra’s Trenza of Her Path Towards Conocimiento. Equity Excell. Educ..

[47] Manzo R.D. (2016). Parent involvement practices of farm working immigrant mothers in a rural community. Assoc. Mex. Am. Educ. J..

